# Next-generation carrier screening: are we ready?

**DOI:** 10.1186/s13073-014-0062-x

**Published:** 2014-08-26

**Authors:** Thomas W Prior

**Affiliations:** Department of Pathology, Ohio State University, Neil Avenue, Columbus, OH 43210 USA

## Abstract

Next-generation sequencing (NGS) methodology allows for a major expansion in current carrier screening tests. NGS testing has been shown to be analytically accurate and cost-effective, but major challenges include educational and counseling issues.

In the molecular diagnostic laboratory, carrier testing is often performed on individuals with a known family history of disease. This type of directed carrier testing is relatively straightforward because there is a known history of the disorder and because the causative mutation has often been identified in the proband. The goal of population-based carrier screening is to identify couples who are at risk of having a child with the disorder without the need for a family history, thus allowing carriers to make informed reproductive choices. It is generally accepted that the following five criteria should be met in order for a screening program to be successful: first, the disorder is clinically severe; second, there is a high frequency of carriers in the screened population; third, a reliable test with a high specificity and sensitivity that is relatively inexpensive is available; fourth, prenatal diagnosis is available; fifth, there is access to genetic counseling.

Regardless of the reliability of the technology used for detecting carriers, an educational component including formal genetic counseling services is absolutely essential. It is important that all individuals undergoing testing understand that a carrier is a healthy individual who is not at risk of developing the disease, but who has a risk of passing the gene mutation on to his/her offspring. Counseling must also include a description of the disorder, therapeutic strategies, and prognosis. Educational material about the disorder should also be made available to all couples, preferably in the pre-conception period, so that they can understand the limitations of the testing. All identified carriers should be referred for follow-up genetic counseling that includes a discussion of risk to the fetus or future pregnancies, and prenatal or pre-implantation diagnosis should be offered. As is true for all carrier screening programs, the testing is voluntary. Informed consent and the usual caveats, including issues relating to confidentiality, paternity, discrimination, self-esteem and cost, must be addressed. Last, before widespread adoption of a carrier-screening program, prospective pilot studies should be performed. The results of pilot studies often help researchers to project outcomes and to complete a realistic national picture of what might be expected from large-scale programs.

## Brief history

Carrier screening for those without a family history was initially confined to screening for genetic disorders that were known to occur with high frequencies in specific ethnicities. The first genetic disorder for which systematic carrier screening was undertaken was Tay-Sachs disease, which has increased prevalence in Eastern European Jewish (Ashkenazi) individuals. Tay-Sachs disease serves as a model disease for carrier screening as it meets all the criteria described earlier. It is an autosomal recessive neurodegenerative disorder that presents in the first year of life and is fatal in early childhood. There is no effective treatment available.

The disease is caused by a mutation in the gene that encodes β-hexosaminase A (HexA), which results in the accumulation of a glycolipid, the ganglioside Gm2, within the lysosome. This leads to blindness, loss of neurologic function and death between the ages of 2 and 4 years. In the Ashkenazi population, Tay-Sachs disease has a very high incidence of about 1:3,000 and a carrier frequency of 1:30. Screening is based on a biochemical assay that measures the activity of Hex A. DNA testing for the three most common mutations that are associated with the disease has been suggested as an alternative to biochemical testing, as 93 to 99% of carriers in the Ashkenazi population will be positive for one of the mutations. The screening has been extremely successful and has led to a reduction of approximately 90% in disease incidence [1]. Today, owing to the success of the Tay-Sachs screening program, testing for Jewish genetic diseases has been expanded to include a number of additional disorders [2].

Cystic fibrosis (CF) is an autosomally recessive inherited disorder that is characterized by the classic triad of obstructive pulmonary disease, pancreatic insufficiency and elevated chloride levels in sweat. The disease is caused by mutations in the gene that encodes the cystic fibrosis transmembrane regulator (CFTR). More than 1,900 mutations have been identified in the gene. CF has a carrier frequency of about 1 in 25 in individuals of northern European descent, but occurs at a lower frequency in other ethnic groups. Since 2001, in response to data from several funded CF carrier screening pilot projects, the American College of Obstetricians and Gynecologists (ACOG) and the American College of Medical Genetics (ACMG) have recommended offering CF carrier screening to all pregnant women [3].

Both the ACOG and the ACMG recommend use of a 23-mutation panel, which tests for all CF-associated mutations with an allele frequency of at least 0.1% in the general US population. Although carrier testing for Tay-Sachs disease among the Ashkenazi population has a test sensitivity of nearly 100%, CF screening using the 23-mutation *CFTR* panel has a lower sensitivity of approximately 80% in Northern Europeans. Several laboratories offer testing for more mutations (expanded panels) than are screened for in the testing recommended by ACMG or ACOG. The benefit of expanded panels is uncertain. A panel that can identify the presence or absence of additional mutations only provides a marginal increase in sensitivity in identifying CF carriers, and the genotype-phenotype association is not well understood because of the rarity of some of the additional mutations. A recent systematic review demonstrated that population-based CF carrier testing was associated with a relatively high uptake, positive attitudes, correct recall and understanding of carrier status, and no long-term psychological harm [4]. Overall the implementation of carrier screening has also been shown to be associated with a modest reduction in the incidence of CF [4,5]. A time-related decrease in birth rate, with a mean annual frequency of 0.16 (95% confidence interval, 0.09 to 0.23) per 10,000 neonates, was reported by Castellani *et al*. [5].

## The future: next-generation sequencing

In the past decade, the cost of DNA sequencing has been greatly reduced due to the emergence of next-generation sequencing (NGS) technologies. The major advance offered by NGS is the ability to produce an enormous volume of DNA sequence inexpensively, allowing the rapid and efficient testing of many genes and hundreds of mutations concurrently. Furthermore, not only have NGS assays been shown to be extremely robust but they are also reproducible and accurate [6]. NGS has facilitated the accurate molecular diagnosis of clinically complex and genetically heterogenous genetic disorders. NGS using gene panels is currently being used for cancer, cardiomyopathy and autism screening.

Thus, pan-ethnic population carrier screening by NGS for a large number of genetic disorders has now become technically feasible. NGS carrier screens not only allow the expansion of the number of mutations tested for, and therefore the identification of rarer mutations, but also make it possible to increase greatly the number of genes screened for that are associated with a number of different diseases. Utilizing NGS, Bell *et al*. [7] performed carrier screening for 448 severe recessive disorders of childhood. Although the technology has been shown to be analytically accurate and reproducible, it presents a challenge that becomes more clinical in nature. The factors that must be taken into account when performing massive sequencing of healthy patients who wish to know their carrier status and reproductive risk are different from those that concern symptomatic patients who require a molecular diagnosis by NGS. A major difference is that the carrier testing should be accompanied by both pre-test and post-test counseling to ensure that the individuals are sufficiently informed of the implications of a positive or a negative carrier test. Also, one issue that must be addressed prior to recommending pan-ethnic carrier screening is the development of educational materials as this becomes a major challenge when testing for a large number of genes associated with a number of different diseases. Prospective pilot studies should also be completed in order to determine which genes should be included in the panels, as well as patient preference and best practices relating to patient counseling.

A screening program that is based on the DNA sequencing of many genes will reveal not only pathogenic mutations but also sequence variations of uncertain significance. Whereas traditional carrier screening is designed to test for the most common known mutations, NGS panels are designed to test for all sequence changes. Such sequence variations can pose difficulties for physicians and genetic counselors when trying to convey accurate results to a potential carrier. One of the mutations on the initial CF panel was later found to be a polymorphism and was removed from the original screening panel [8]. The carrier-screening study by Bell *et al*. [7] found that 27% of the mutations cited in the literature as being associated with disease were polymorphisms or misannotated. Given the large number of sequence variations that are likely to be generated when screening a large number of genes, the time and effort required to establish causality can be significant. Also many of the true causative mutations identified will be very rare or novel with little known about their clinical or prognostic impacts.

NGS carrier screening assays must be rigorously validated, both analytically and clinically, before entering the clinical laboratory. Although the cost of multiplexing a number of genes simultaneously by NGS is much less than analyzing those genes individually, the additional costs of interpretation of the results, reporting and genetic counseling that result from NGS must be recognized and dealt with. Most importantly, the data generated will assist couples in making reproductive decisions relating to a large number of severe recessive disorders. The number and choice of genetic tests available to patients will continue to increase. There is no question that education for both patients and physicians is a priority for a successful carrier screening program; it is imperative that the results be conveyed to the patient in a comprehensive and efficient manner.

## Abbreviations

ACMG: American College of Medical Genetics
ACOG: American College of Obstetricians and Gynecologists
CF: cystic fibrosis
CFTR: cystic fibrosis transmembrane regulator
Hex A: β-hexosaminase A
NGS: next-generation sequencing
